# Bioinformatic comparison of Kunitz protease inhibitors in *Echinococcus granulosus* sensu stricto and *E. multilocularis* and the genes expressed in different developmental stages of *E. granulosus s.s.*

**DOI:** 10.1186/s12864-021-08219-4

**Published:** 2021-12-18

**Authors:** Hui Zhang, Mengxiao Tian, Wenjing Qi, Juan Wu, Huajun Zheng, Gang Guo, Liang Zhang, Shiwanthi L. Ranasinghe, Donald P. McManus, Jun Li, Wenbao Zhang

**Affiliations:** 1 Basic Medicine College, Xinjiang Medical University, 830011 Urumqi, Xinjiang China; 2 State Key Laboratory of Pathogenesis, Prevention and Treatment of High Incidence Diseases in Central Asia, WHO-Collaborating Centre for Prevention and Care Management of Echinococcosis, Xinjiang Medical University, The First Affiliated Hospital of Xinjiang Medical University, 830054 Urumqi, Xinjiang China; 3 Shanghai–Ministry of Science and Technology Key Laboratory of Health and Disease Genomics, Chinese National Human Genome Center at Shanghai, Shanghai, China; 4 Molecular Parasitology Laboratory, Infectious Diseases Program, QIMR Berghofer Medical Research Institute, Brisbane, Queensland Australia

**Keywords:** Kunitz inhibitors, *E. multilocularis*, *E. granulosus* *sensu stricto*, Bioinformatic analysis, Stage expression

## Abstract

**Background:**

Cystic and alveolar echinococcosis caused by the tapeworms *Echinococcus granulosus* sensu stricto (*s.s.*) and *E. multilocularis*, respectively, are important zoonotic diseases. Protease inhibitors are crucial for the survival of both *Echinococcus spp*. Kunitz-type inhibitors play a regulatory role in the control of protease activity. In this study,we identified Kunitz-type domain protease inhibitors(KDPIs) present in the genomes of these two tapeworms and analyzed the gene sequences using computational, structural bioinformatics and phylogenetic approaches to evaluate the evolutionary relationships of these genes. Hi-seq transcriptome analysis showed that *E. granulosus* *s.s.* KDPIs were differentially expressed in the different developmental stages. We validated some of the genes expressed in adult worm, protoscolex and cyst germinal membrane of *E. granulosus* *s.s.* and *E. multilocularis* by quantitative PCR.

**Results:**

A total of 19 genes from *E. multilocularis* and 23 genes from *E. granulosus* *s.s.* were predicted to be KDPIs with the most containing a single Kunitz-domain. A maximum likelihood method phylogenetic tree indicated that the *E. granulosus* *s.s.* and *E. multilocularis* Kunitz domain peptides were divided into three branches containing 9 clusters. The ratio of positively charged residues and neutral residues are different between *E. multilocularis* and *E. granulosus* *s.s.* KDPIs. We also found that *E. multilocularis* had higher percentage of sequences containing signal peptides (17/19, 89.47%) than that of *E. granulosus* *s.s.* (14/23, 60.87%). Transcript analysis showed all the *E. granulosus* *s.s.* KDPI genes were expressed differentially in four developmental stages of the worm. Transcription analysis showed that 9 KDPIs (including EG_07244,EGR_08716 and EGR_10096) were highly upregulated in adult worm, and 2 KDPIs (EG_09268 and EG_09490) were highly expressed in the cyst germinal membrane. Quantitative gene expression analysis(qPCR) of four genes confirmed the expression of these genes. EGR_08716 and its homologous gene (EmuJ_001137000) were highly and specifically expressed in adult worms of the two worms.

**Conclusions:**

A total 19 and 23 KDPIs were identified in the genomes of *E. multilocularis* and *E. granulosus s.s. *, respectively. The differential expression of these KDPIs in different stages may indicate their different roles in the different hosts. The difference in characterization of KDPIs may be associated with the different pathology of metacestode stage of these two parasites.

**Supplementary Information:**

The online version contains supplementary material available at 10.1186/s12864-021-08219-4.

## Background

Cyst echinococcosis (CE) and alveolar echinococcosis (AE) are both medically and economically important diseases caused by the metacestode stages of *Echinococcus granulosus* sensu stricto (*s.s.*)and *E. multilocularis* respectively. The diseases impact on hundreds of millions of people in Asia, Europe, American and Africa [1]. The control and treatment of echinococcosis are difficult. High frequency of dosing dogs with the drug praziquantel has played a key role in the control of the disease [2, 3], but undertaking the control measure is challenging in remote areas. A vaccine against adult worms in dogs is urgently needed [4].

The life-cycle of these two tapeworms involves four developmental stages including adult worm, oncosphere, cyst and protoscolex present in their definitive and intermediate hosts. The major definitive and intermediate hosts of *E. granulosus* *s.s.* are dogs and sheep respectively, whereas, the natural definitive and intermediate hosts of *E. multilocularis* are fox/wolf and rodent small mammals respectively. Humans are the intermediate hosts of these two tapeworms. The survival of these tapeworms relies on evading host immune responses and avoiding attack by proteases; this is especially important for the adult parasites which reside in the gastrointestinal duct, a location where high concentration of proteases are present which are harmful and toxic for the worms.

Eukaryote proteases including serine (trypsin/chymotrypsin-like), cysteine (thiol) and aspartic (pepsin/cathepsin/rennin) proteases play a fundamental role in the regulation of protein function. Their functions and activities are controlled largely by protease inhibitors which play crucial roles in the regulation of proteases involved in a range of biological processes system including cell proliferation, inflammation, cell homeostasis and immune mechanism [5–7]; protease inhibitors act mainly through the control of potentially disadvantageous, excessive or inopportune proteolytic activity. Protease inhibitors including aspartic, cysteine, metallo, serine, and threonine inhibitors are super-families based on their similarities at the amino acid sequence level and tertiary structure [8]. Similarities in primary structure and tertiary structure support the common ancestry of many protease inhibitor families.

Kunitz-type domain protease inhibitors (KDPIs) are an important type of protease inhibitor and belong to the I2 family of protease inhibitors [8, 9]. These inhibitors contain at least one cysteine-rich peptide chain (Kunitz-type domain) with α and β sheets. The Kunitz domain consists of around 60 amino acids including six conserved cysteine residues forming three disulphide bridges in a characteristic pattern (C1-C6, C2-C4, and C3-C5) [9, 10], which stabilize these inhibitors. KDPIs have been characterized from animals and plants [9, 11] including helminths [12–14]. A previous study described eight genes (EgKU1-EgKU8) isolated from *E. granulosus* *s.s.* protoscoleces treated with pepsin/H (+) [15]. We previously cloned and characterized two *E. granulosus* *s.s.* KDPIs, EgKI-1(EG_08721 (GenBank: EUB56407.1)) and EgKI-2 (EG_07242 (GenBank: EUB57880.1)) [14]. EgKI-1 is highly expressed in the oncosphere (egg) stage and is a potent chymotrypsin and neutrophil elastase inhibitor that binds calcium and reduced neutrophil infiltration in a local inflammation model. EgKI-2 is highly expressed in adult worms, it is a potent inhibitor of trypsin and is a potential vaccine candidate against echinococcosis in dogs [14]. Beyond these, other *E. granulosus* *s.s.* and *E. multilocularis* KDPIs have been received little attention.

In the present study, we identified all KDPI sequences predicted in the *E. granulosus* *s.s.* and *E. multilocularis* genomes and used computerized programs to characterize these Kunitz domain protease inhibitors. We show that the majority of *E. granulosus* *s.s.* KDPIs were differentially expressed in different life cycle stages and some have a range of GO numbers indicating these inhibitors likely function in different ways in the tapeworm’s development.

## Results

### General characterizations of Kunitz domain protease inhibitors

**Fig. 1:**
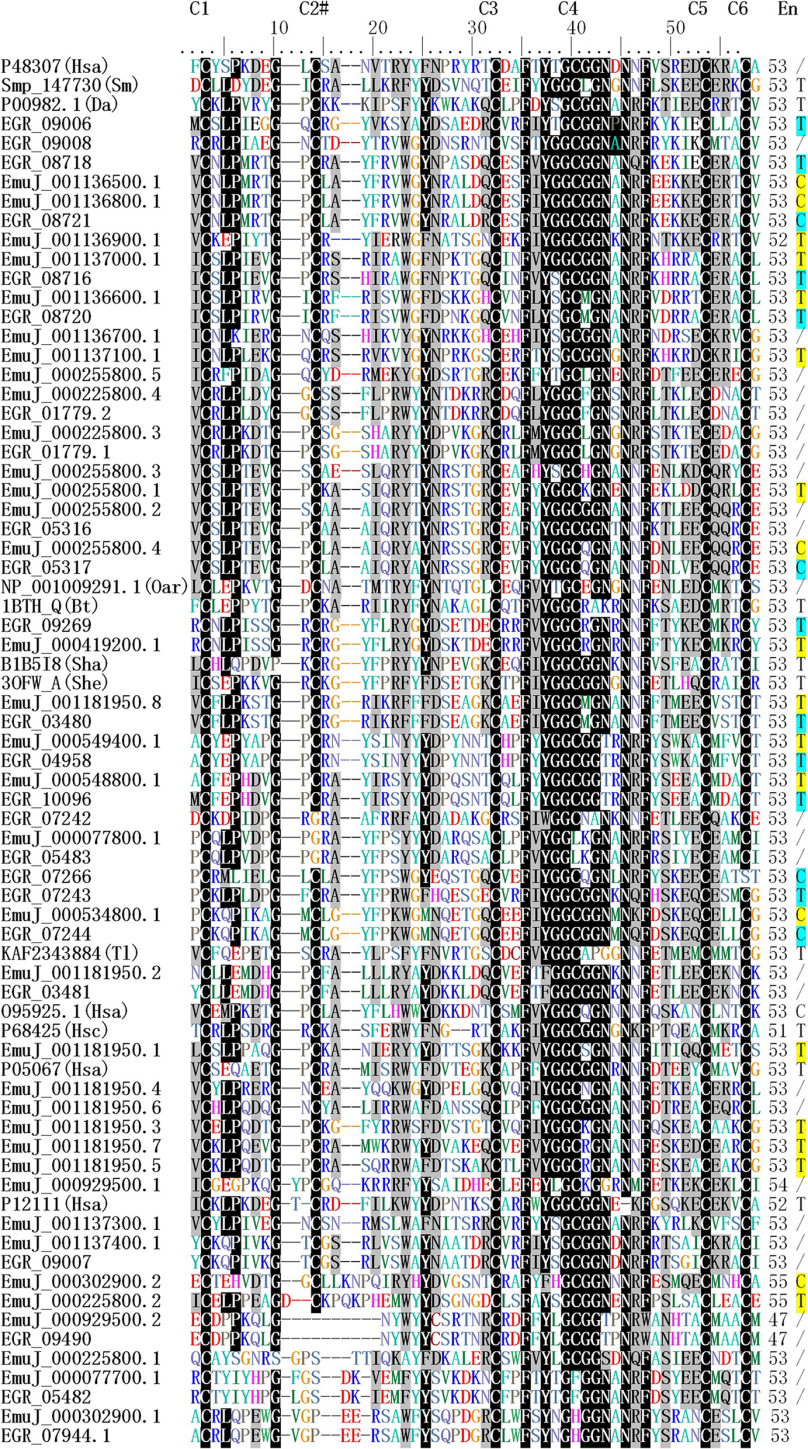
Alignment and clustering of *E. multilocularis* and *E. granulosus*  *s.s.* Kunitz-type domain protease inhibitors. These inhibitor protein sequences are compared with the homologue sequences (blocked in gray background) *Homo sapiens* including P48307(Hsa), P05067(Hsa), P12111(Hsa) and O95925(Hsa), *Ovis aries* (NP_001009291.1(Oar)), *Bos taurus*(1BTH_Q(Bt)), *Schistosoma mansoni* (Smp_147730 (Sm)), *Stichodactyla haddoni*(B1B5I8(Sha)), *Stichodactyla helianthus* (3OFW_A(She)), *Trinorchestia longiramus*(KAF2343884(Tl)), *Haplopelma schmidti* (P68425(Hsc)). “En”, “T”, “C” and “/” represent enzyme and inhibitor of trypsin, chymotrypsin, and nonprediction, respectively

InterproScan and Motif scan identified 19 and 23 genes encoding KDPIs from the *E. multilocularis* and *E. granulosus* *s.s.* genomes, respectively (Table 1). The KDPI family has a typical Kunitz domain of about 60 amino acids in size (Fig. 1) with a special secondary structure formed by three disulphide bonds or bridges (Additional file 3: Fig. S1). The echinococcal Kunitz domains contain an average of 52.85 aa (range 47-55 aa) with the majority comprising 53 aa (Fig. 1).

**Table 1:** Summary of physiological and biological characteristics of Kunitz protease inhibitors in *E. multilocularis* and *E. granulosus s.s. *

Species	*E. multilocularis*	*E. granulosus s.s*
KDPIs/single KDPIs	19/16	23/21
Number of amino acids	333.47	269.96
Molecular weight (Da)	37153.2	30117.02
Isoelectric points	7.44	7.84
No. of trans-domain (%)	4(21.05)	5(21.74)
No. of cysteine in Kunitz domain	5.63	5.65
No. of cysteine in the protein	24.89	18.13
Instability index	45.17	47.36
Stable protein (No/yes)	12/7	15/8
Aliphatic index	72.35	71.37
Grand average of hydropathicity	-0.22	-0.22
Signal peptides (%)	17(89.47) ^*^	14(60.87)
No. of Kunitz Motifs	1.79	1.17
En-t-In(T/C)	9/2	9/4

Note: KDPIs, Kunitz domain protease inhibitors; No. of aa, number of amino acids; No. of tran-domain, percentage of containing transmembrane domains; Aver of cysteine, average of cysteine per sequence; Aliphatic indexes; GRSVY, hydropathic index; En-t-In (T/C), enzyme targeting inhibitors, trypsin inhibitors(T) or chymotrypsin inhibitors(C)

There are significant differences of signal peptides between *E. multilocularis* and *E. granulosus*  *s.s.* KDPIs (χ^2^ = 13.544, *P* < 0.01)

Among these KDPIs, *E. multilocularis* has 16 KDPIs containing a single Kunitz domain with the complete proteins having sizes of 75-534 aa. There are 3 proteins containing multi-domains with a maximum 8 Kunitz domains (EmuJ_001181950) in size ranging from 610 to 2425 aa. *E. granulosus*  *s.s.* has 21 single Kunitz domain KDPIs of 75-976 aa in size, and 2 multiple domain KDPIs sized from 878 to 1540 aa. The molecular weights of these KDPIs range from 8.34 kDa to 266.77 kDa with isoelectric points from 4.52 to 10.52 (Additional file 1: Table S1). The majority of single Kunitz domain proteins comprise less than 100 amino acid (Additional file 1: Table S1).

We used the instability index to estimate the stability of the KDPIs. An instability value >40 is an unstable protein. The index value representing rigidity/flexibility of each peptide varied (26.51-94.41). The average value was 45.17 for *E. multilocularis* and 47.37 for *E. granulosus  s.s. *, suggesting the peptides may be flexible. The analysis showed that *E. multilocularis* has 12 unstable and 7 stable KDPIs and *E. granulosus*  *s.s.* has 15 unstable and 8 stable KDPIs (Additional file 1: Table S1).

Sequence analysis showed that these echinococcal KDPI sequences contain a high percentage of hydrophobic residues including alanine (A), valine (V), leucine (L) and isoleucine (I). The hydropathic index (Grand Average of Hydropathy: GRAVY) for *E. granulosus*  *s.s.* and *E. multilocularis* are -0.223 ± 0.333 and -0.222 ± 0.312, respectively. Aliphatic indexes (AI) are 71.37 ± 14.08 and 72.36 ± 13.75 for *E. granulosus*  *s.s.* and *E. multilocularis*, respectively (Table 1 and Additional file 1: Table S1).

The average numbers of negatively charged residues (Asp+Glu) are 38.42 and 28.91, accounting for 8.84% and 9.13% of *E. multilocularis* and *E. granulosus*  *s.s.* KDPIs, respectively. There are 35.16 and 30.70 positively charged residues (Arg+Lys) in the *E. multilocularis* and *E. granulosus* *s.s.* KDPIs accounting for 12.73% and 12.23% of the total amino acids. Neutral amino acid residues are 259.89 and 210.35 aa on average and account for 78.42% and 78.63% of the KDPIs in *E. multilocularis* and *E. granulosus  s.s. *, respectively (Table 1 and Additional file 1: Table S1).

The average aliphatic indexes are 72.36 (51.54-89.78) and 71.37 (49.44-100.73) for the *E. multilocularis* and *E. granulosus*  *s.s.* KDPIs, respectively. The hydropathicity indexes of *E. multilocularis* and *E. granulosus* *s.s.* KDPIs are -0.222 (ranging from -0.978 to 0.340) and -0.223 (ranging from -0.996 to 0.371) for respectively. The results indicate that the KDPIs in both parasites are likely hydrophilic proteins (Table 1 and Additional file 1: Table S1).

*E. multilocularis* and *E. granulosus*  *s.s.* have 4 and 5 KDPIs containing transmembrane regions, respectively, and 78.94% and 78.26% of the *E. multilocularis* and *E. granulosus* *s.s.* KDPIs are extracellular (Table 1), which matches the GO analysis (Table 2 and Additional file 2: Table S2), indicating that the most KDPIs may involve host and parasite interface responses. The TopPred program indicated that 4 *E. multilocularis* and 5 *E. granulosus*  *s.s.* KDPIs are located on the cytoplasm (Additional file 1: Table S1) with others, including 15 Em-KDPI sequences and 18 Eg-KDPIs, being extracellular.

**Table 2:** The expression of Kunitz-type domain protease inhibitors in four stages of *E. granulosus*  *s.s.* showing Hi-seq reads

Gene ID	Adt	Onc	PSC	CGM	Seq. Description	Seq. Lth	#GOs	GOs
EGR_07244	217	0	1	10	serine protease inhibitor	106	1	F: serine-type endopeptidase inhibitor activity
EGR_08716	155	0	0	0	kunitz-type protease inhibitor 3-like	84	3	F: peptidase inhibitor activity; F: protein binding; P: transforming growth factor beta receptor signaling pathway
EGR_10096	70	0	3	4	kunitz domain-containing	98	42	C: cytoplasmic vesicle; P: apoptosis; P: neuromuscular process controlling balance; P: ionotropic glutamate receptor signaling pathway; P: regulation of epidermal growth factor receptor activity; F: receptor binding; P: regulation o
EGR_07242	46	0	1	5	serine protease inhibitor	83	7	F: peptidase inhibitor activity; C: nematocyst; F: ion channel inhibitor activity; F: potassium channel inhibitor activity; F: serine-type endopeptidase inhibitor activity; C: extracellular region; P: pathogenesis
EGR_07243	35	0	2	5	wap four-disulfide core domain 6b	75	2	C: cytoplasm; F: serine-type endopeptidase inhibitor activity
EGR_03480	24	0	1	5	four-domain proteases inhibitor	88	1	F: peptidase inhibitor activity
EGR_09490	17	2	14	51	spon-1 protein	976	3	F: serine-type endopeptidase inhibitor activity; C: proteinaceous extracellular matrix; C: extracellular region
EGR_03481	16	0	8	2	trypsin inhibitor	242	4	F: peptidase inhibitor activity; P: multicellular organismal process; F: extracellular matrix structural constituent; C: proteinaceous extracellular matrix
EGR_08720	10	0	0	0	kunitz bovine pancreatic trypsin inhibitor domain protein	84	4	F: serine-type endopeptidase inhibitor activity; F: peptidase activity; F: peptidase inhibitor activity; C: extracellular region
EGR_07944	9	0	22	14	kunitz bovine pancreatic trypsin inhibitor domain protein	539	2	C: extracellular region; F: hydrolase activity
EGR_09269	8	0	0	1	tissue factor pathway inhibitor 2-like	92	2	F: extracellular matrix structural constituent; C: proteinaceous extracellular matrix
EGR_05317	7	0	19	16	Kunitz-like protease inhibitor precur	1540		
EGR_08721	6	108	0	0	serine protease inhibitor- with kunitz and wap domains 1	79	1	C: acrosomal vesicle
EGR_04958	5	0	1	2	elegans protein partially confirmed by transcript evidence	135	2	F: serine-type endopeptidase inhibitor activity; P: epidermis development
EGR_05483	5	12	0	2	secreted protein with kunitz	191	2	C: extracellular region; F: hydrolase activity
EGR_09007	4	0	0	0	mechanosensory abnormality family member (mec-1)	86	2	P: extracellular structure organization; P: mechanosensory behavior
EGR_09008	3	0	0	0	acp24a4	102	2	C: extracellular region; F: peptidase activity
EGR_01779	1	0	13	3	isoform g	878	1	F: hydrolase activity
EGR_05482	1	2	0	0	kunitz bovine pancreatic trypsin inhibitor domain containing protein	130	1	C: extracellular region
EGR_07266	1	0	0	3	serine protease inhibitor	129	3	F: binding; F: serine-type endopeptidase inhibitor activity; C: extracellular region
EGR_05316	0	0	3	2	kunitz-type protease inhibitor 3-like	239	1	F: peptidase inhibitor activity
EGR_08718	0	2	0	0	kunitz domain-containing	144	4	F: peptidase activity; F: serine-type endopeptidase inhibitor activity; F: peptidase inhibitor activity; C: extracellular region
EGR_09006	0	0	0	0	single kunitz protease inhibitor	89	5	F: peptidase activity; F: serine-type endopeptidase inhibitor activity; F: peptidase inhibitor activity; C: extracellular region; P: multicellular organismal development

Note: Adt, adult worms; Onc, oncospheres; PSC, protoscoleces; CGM, Cyst Germinal membrane

Signal peptide analysis showed that there are 17/19(89.47%) *E. multilocularis* KDPIs having an 18-26 amino acid (aa) signal peptide and 2/19(10.53%) KDPIs without. In contrast, *E. granulosus*  *s.s.* has 14/23 (60.87%) KDPIs containing signal peptide sequences and 9/23 (39.13%) KDPIs without (Table 1). There is significant difference of signal peptides between *E. multilocularis* and *E. granulosus*  *s.s.* KDPIs (χ^2^ = 13.544, *P* <0.01, Table 1).

### Cluster and phylogenetic analysis of Kunitz protease inhibitors

**Fig. 2:**
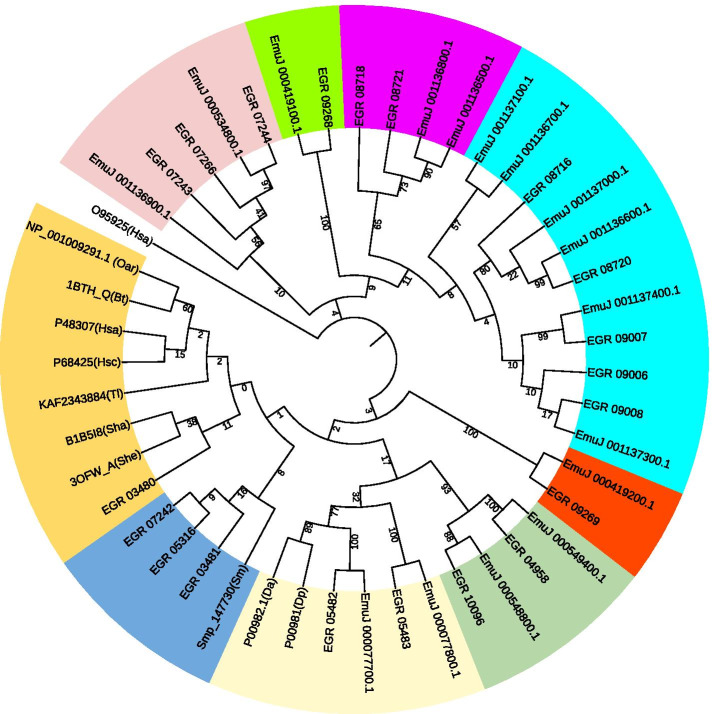
Phylogram constructed to compare the sequences of *E. granulosus  s.s.*- and *E. multilocularis*-KDPIs with KDPIs from *Bos taurus*, *Homo sapiens* and other species. These inhibitor protein sequences are also compared with the sequences from *Dendroaspis* including *Dendroaspis angusticeps* (P00982.1(Da)), *Dendroaspis polylepis* (P00981(Dp)). The phylogenetic tree was constructed using MEGA 7.0 and Interactive Tree of Life iTOL v6 with the maximum likelihood method. Bootstrapping analysis was performed and the bootstrap values from 1000 replicate iterations are shown on the nodes. The clades are represented by different color

Multiple sequence alignment (Fig. 1) and phylogenetic analysis (Fig. 2) of the amino acid sequences were used to infer the evolutionary relationships between the *E. multilocularis* and *E. granulosus*  *s.s.* KDPIs and to make a comparison with other species. Figure 2 shows the different evolutionary distances of the genes containing single Kunitz domain of the KDPIs using the maximum likelihood method. The maximum likelihood tree indicated that these echinococcal KDPIs were divided into three branches with 9 clusters. One branch contains several Echinococcus KDPIs such as EGR_3480, EGR_3481, EGR_05316 and EGR_07242 are close to the KDPIs from human, sheep, cattle and other species (Fig. 2 and Additional file 6: Fig. S4). Whereas other two branches are relatively *Echinococcus* unique (Fig. 2).

It showed that two KDPIs of EGR_05482 and EmuJ_000077700.1 from *E. granulosus*  *s.s.* and *E. multilocularis* shared a high homology with mamba venom toxin P00982.1 and P00981 from *Dendroaspis angusticeps* and *Dendroaspis polylepis* respectively (Fig. 2), indicating these echinococcal KDPIs may have similar pathophysiological functions of mamba venom blocking ion channels and membrane receptors.

### Comparison of KDPI genes predicated from the *E. granulosus*  *s.s.* and *E. multilocularis *genomes

We compared the KDPI genes predicted from the genomes of *E. granulosus*  *s.s.* and *E. multilocularis* and found that some genes are species-specific. *E. multilocularis* does not have homologues of *E. granulosus*  *s.s.* sequences EG_07242, EG_07266, EG_07243, EG_09006 and EG_09008; whereas EmuJ_001136700.1 and EmuJ_001137100.1 are specific to *E. multilocularis* (Additional file 5: Fig. S3).

The specificity of a protease inhibitor against a protease is mainly determined by the nature of the amino acid residue at position P1 of its active site. It has been shown that Lys(K) and Arg(R) mutants of bovine pancreatic trypsin inhibitor (BPTI) bind to bovine trypsin about 10^5^-fold stronger than BPTI with P1 Tyr(T) [16]. In addition, it has been shown that typical trypsin inhibitors have Arg(R) or Lys(K) at P1, and chymotrypsin inhibitors have Leu (L) or Met (M) at the P1 position [17]. Therefore, the sequence analysis shows that the *E. multilocularis* has 10 KDPIs containing R at P1 and *E. granulosus*  *s.s.* has 11 KDPIs containing R at P1, which belong to typical trypsin inhibitors. Furthermore, the two tapeworms have 3 or 4 sequences containing L at P1 respectively, which are chymotrypsin inhibitors (Fig. 1; Table 1).

### 2 D and 3 D structure of Kunitz domain protease inhibitors

The majority of *E. multilocularis* and *E. granulosus*  *s.s.* single KDPIs are small proteins sized 16-kDa and contain a relatively high percentage of Lys and Arg residues at the C-terminus. Like most Kunitz domain protease inhibitors, the Em- and Eg-KDPIs contain a conserved Kunitz type sequence with 6 cysteine residues forming three disulphide bridges (C1-C6, C2-C4 and C3-C5) (Additional file 3: Fig. S1) and these bridges play a key role in the formation of the 2D and 3D structure of these KDPIs. For the single Kunitz domain sequences, the secondary structure prediction revealed 19.01-52.71% and 18.6-60.35% of α-helix and random coil structures in Eg-KDPIs, followed by extended strands and β-turn structure, accounting for 13.1-26.67% and 1.89-10.84% respectively. Em-KDPIs α-helix and random coil structures account for 19-40.45% and 32.5-55.99% of the protein sequence respectively, followed by extended strands and β-turn, accounting for 8-36.25% and 0-10.71% (Table 3).

**Table 3:** The secondary structure prediction of the single Eg-KDPIs and Em-KDPIs

Accession number	Alpha helix(aa/%)	Extended strand(aa/%)	Beta turn(aa/%)	Random coil(aa/%)
EGR_03480	32/36.36	19/21.59	3/3.41	34/38.64
EGR_03481	46/19.01	57/23.55	19/7.85	120/49.59
EGR_04958	33/24.44	28/20.74	11/8.15	63/46.67
EGR_05316	92/38.17	54/22.41	17/7.05	78/32.37
EGR_05482	54/41.54	18/13.85	8/6.15	50/38.46
EGR_05483	66/34.55	41/21.47	10/5.24	74/38.74
EGR_07242	25/30.12	13/15.66	9/10.84	36/43.37
EGR_07243	20/26.67	20/26.67	5/6.67	30/40
EGR_07244	41/38.68	23/21.7	2/1.89	40/37.74
EGR_07266	68/52.71	29/22.48	8/6.2	24/18.6
EGR_07944.1	138/25.6	91/16.88	34/6.31	276/51.21
EGR_08716	31/36.9	11/13.1	3/3.57	39/46.43
EGR_08718	41/28.47	34/23.61	8/5.56	61/42.36
EGR_08720	25/29.76	18/21.43	4/4.76	37/44.05
EGR_08721	35/44.3	13/16.46	3/3.8	28/35.44
EGR_09006	28/31.46	22/24.72	9/10.11	30/33.71
EGR_09007	23/26.74	13/15.12	7/8.14	43/50
EGR_09008	43/42.16	22/21.57	7/6.86	30/29.41
EGR_09269	37/40.22	15/16.3	4/4.35	36/39.13
EGR_09490	203/20.8	145/14.86	39/4.0	589/60.35
EGR_10096	20/20.41	23/23.47	3/3.06	52/53.06
EmuJ_000077700.1	65/30.95	42/20	17/8.1	86/40.95
EmuJ_000077800.1	33/24.26	39/28.68	10/7.35	54/39.71
EmuJ_000302900.1	128/23.97	76/14.23	31/5.81	299/55.99
EmuJ_000419200.1	37/40.22	15/16.30	0/0.00	40/43.48
EmuJ_000534800.1	24/32.00	6/8.00	5/6.67	40/53.33
EmuJ_000548800.1	23/23.23	21/21.21	4/4.04	51/51.52
EmuJ_000549400.1	19/19.00	27/27.00	8/8.00	46/46.00
EmuJ_001136500.1	36/40.45	16/17.98	2/2.25	35/39.33
EmuJ_001136600.1	31/36.90	13/15.48	6/7.14	34/40.48
EmuJ_001136700.1	20/25.64	18/23.08	8/10.26	32/41.03
EmuJ_001136800.1	36/40.45	16/17.98	2/2.25	35/39.33
EmuJ_001136900.1	21/23.33	17/18.89	4/4.44	48/53.33
EmuJ_001137000.1	26/30.95	16/19.05	4/4.76	38/45.24
EmuJ_001137100.1	22/26.19	20/23.81	9/10.71	33/39.29
EmuJ_001137300.1	20/25	29/36.25	5/6.25	26/32.5
EmuJ_001137400.1	26/30.59	16/18.82	4/4.71	39/45.88

Note: aa/%, number/percentage of amino acids in each secondary structure

**Fig. 3:**
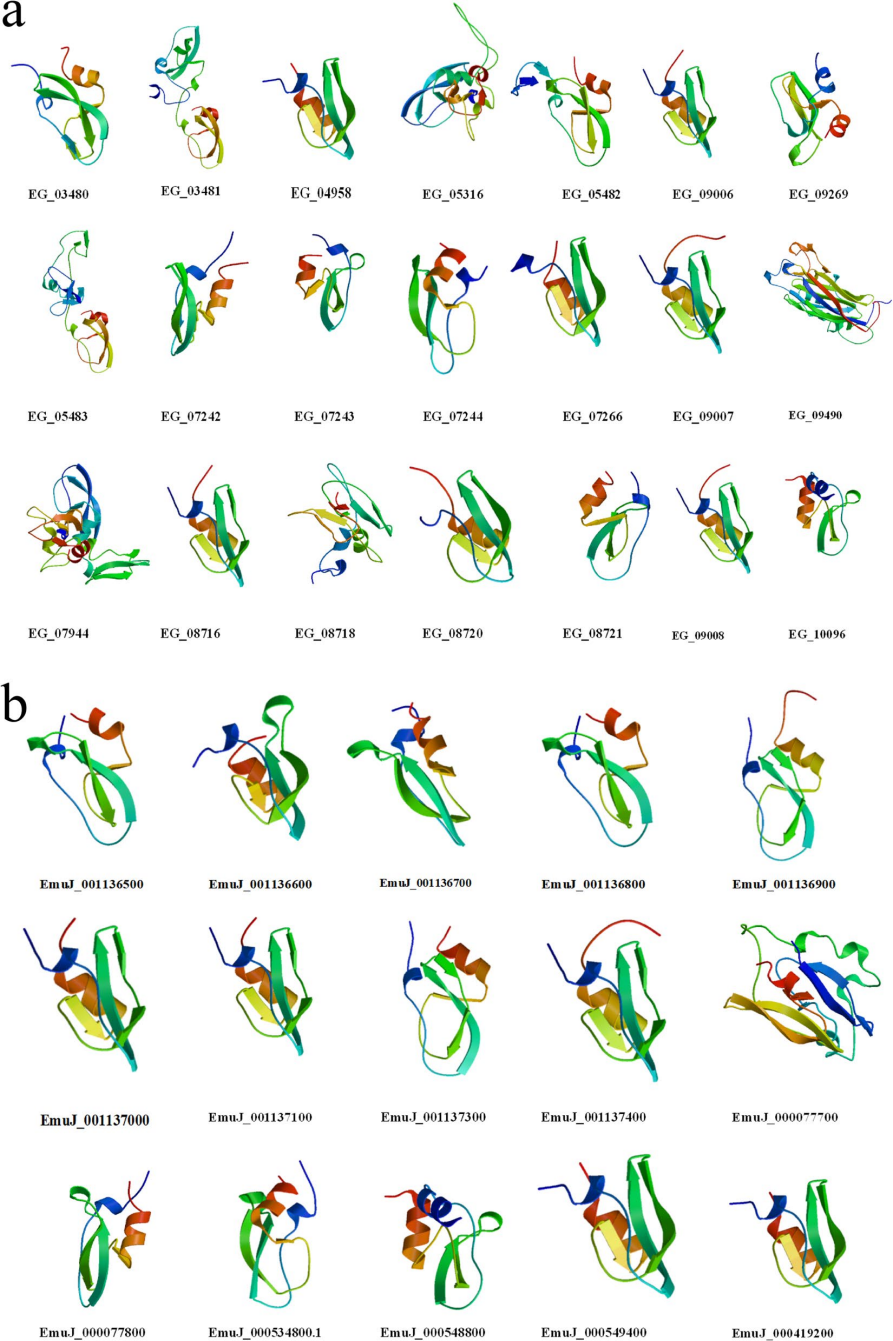
Three dimensional structures of single Kunitz domain protease inhibitors in *E. granulosus*  *s.s.* and *E. multilocularis* using SWISS-MODEL. **a** Structures of single Kunitz domain protease inhibitors in *E. granulosus  s.s. .* **b** Structures of single Kunitz domain protease inhibitors in *E. multilocularis*

It is accepted that there is a close relationship between the structure and function of a protein. Therefore, we used SWISS-MODEL to predict 3D structures based on the homology modeling of KDPI templates from PDB (protein database) including single and multiple Kunitz domain proteins (Fig. 3 and Additional file 4: Fig. S2).

Three-dimensional structure analysis showed that a single Kunitz domain sequence with three disulphide bridges has a similar structure containing an α-helix and random coils with similar structures (Fig. 3). The structure of some single Kunitz domain sequences lose the second cysteine (C2) which may impact the 3D structure of these KDPIs. (Fig. 1; Table 1).

### Expression of *E. granulosus*  *s.s.* KDPIs in different developmental stages

To estimate expression of the KDPIs, Hi-seq techniques were employed to obtain the transcript reads of these genes from total RNA from each of 4 developmental stages of *E. granulosus  s.s. *. The transcript read information was published in a previous paper of ours [18]. The transcriptome analysis showed that these Kunitz peptides were differentially expressed in the different developmental stages of *E. granulosus*  *s.s.* (Table 2). All the inhibitors, except EG_09006, were expressed in one or 4 stages of *E. granulosus*  *s.s.* with some being highly and differentially expressed in one or two stages. Transcription analysis showed that 9 KDPIs including EGR_03480, EGR_03481, EGR_07242, EGR_07243, EG_07244,EGR_08716, EGR_08720, EGR_09269 and EGR_10096 were highly up-regulated in adult worm, and two KDPIs (EG_09268 and EG_09490) were highly expressed in the cyst germinal membrane. Sequence analysis showed that some of these adult worm up-regulated genes are extracellular including EG_03480, EG_07242, EG_08716, EG_08720, EG_09490 and EG_10096, and some are intracellular such as EG_03481, EG_07243 and EG_07244 (Table 2 and Additional file 1: Table S1).

EG_08716 is an extracellular protease inhibitor and has 42 predicted GOs, including cytoplasmic vesicle for neuromuscular process controlling balance, ionotropic glutamate receptor signaling pathway, regulation of the activity of epidermal growth factor receptor and synapse, regulation of mitotic cell cycle and translation and cellular copper and calcium ion homeostasis (Additional file 1: Table S1). The expression analysis indicated that this gene may play an important role in adult worm development and against host protease attack. It is interesting that EG_07244 is predicted having endopeptidase activity, indicating that the protein has two functions, as a peptidase and as a protease inhibitor in adult worms. This needs to be identified. EG_08721 is an extracellular inhibitor and was differentially highly expressed in the oncosphere compared with the other stages, indicating this protease inhibitor plays an important role in oncosphere biology, the only stage for primarily infection and EG_08721 may play an important role in oncosphere against host protease attack which may be a candidate for vaccine development.

Although we activated PSC with pepsin, only three KDPIs (EG_01779, EG_05317 and EG_07944) were slightly elevated in this stage. Importantly, we found that EG_09268 and EG_09490 were highly expressed in the cyst germinal membrane and the proteins expressed by these genes may be potential targets for drug development.

**Fig. 4:**
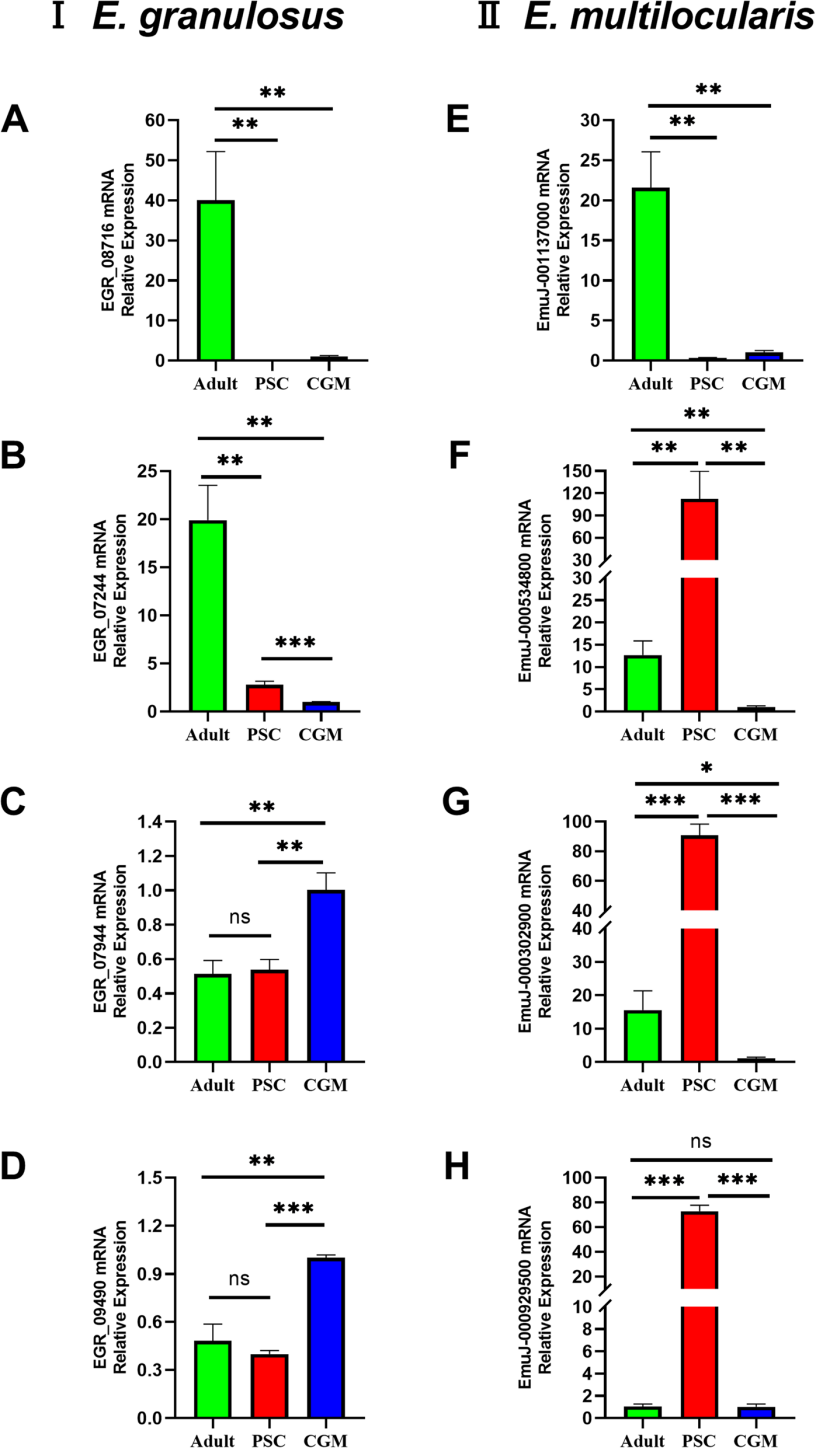
Transcription levels of KDPIs (EGR_08716, EGR_07244, EGR_07944, EGR_09490, EmuJ_001137000, EmuJ_000534800, EmuJ_000302900, and EmuJ_000929500) in different life cycle stages of *E. granulosus*  *s.s.* and *E. multilocularis*. Bars indicate the mean value± standard deviation (SD) of three individual experiments. Differences among different groups were analyzed by one-way ANOVA and Two-tailed Student’s t test. “Adult”, “PSC”, and “CGM” represent adult worm, protoscoleces, and cyst germinal membrane three stages of the tapeworm, respectively

To validate the expression of these genes, a qPCR assay was employed to quantify the transcript levels of 4 genes of *E. granulosus*  *s.s.* (EGR_08716, EGR_07244, EGR_07944 and EGR_09490), which confirmed the expression in cyst germinal membranes, protoscoleces and adult worm stages of the tapeworm. We also identified the homologous KDPI genes expression from *E. multilocularis* (EmuJ_001137000, EmuJ_000534800, EmuJ_000302900, and EmuJ_000929500) in three stages of the tapeworm (Fig. 4). Only one gene, EmuJ_001137000 showed a similar expression pattern as *E. granulosus*  *s.s.* EG_08716, is highly and specifically expressed in the adult worms of *E. multilocularis* (Fig. 4). Other three genes were different from those homologous genes of *E. granulosus  s.s. *, which were highly expressed in protoscoleces of this tapeworm (Fig. 4). The qPCR showed that *E. multilocularis* adult worm EmuJ_001137000 was 4.43 folds higher than EGR_08716 in the adult worms of *E. granulosus  s.s. *.

## Discussion

KDPIs occur in almost all living organisms from bacteria to plants and animals. Kunitz peptides show diverse biological activities including inhibition of proteases and/or blocking or modulating ion channels. Helminth parasites have been reported expression KDPIs [19], such as in *Schistosoma mansoni* [12], *S. japonicum* [20] *Fasciola hepatica* [21, 22], *Ancylostoma caninum* [23], *Ancylostoma ceylanicum* [24], and *Steinernema carpocapsae* [25]. Beside their role in inhibition of proteases such as pancreatic elastase, neutrophil elastase, chymotrypsin and trypsin, KDPIs play an important role in helminth’s immune evasion and development, modulating the inflammatory responses, especially impairing Th1/Th17-associated inflammation response and reduction of neutrophil recruitment [19]. In this study, based on the genomic information available for *E. granulosus* *s.s.* and *E. multilocularis* we identified 23 and 19 KDPIs, respectively. These genes were differential expressed in different developmental stages, indicating these KDPIs may play different role in both parasite development and regulation the interface responses between parasites and hosts.

A remarkable difference between the larval stages of *E. multilocularis* and *E. granulosus* *s.s.* is the difference in the lesion pathology in the intermediate hosts. The metacestode of *E. multilocularis* is a tumor-like, infiltrating structure consisting of many small vesicles embedded in the stroma of connective tissue. The continual growth of parasite vesicles in a proliferative style causes damage of liver tissues, which results in a high mortality of AE patients. In contrast, *E. granulosus* *s.s.* cysts develop in internal organs (mainly liver and lungs) of humans and other intermediate hosts as unilocular fluid-filled bladders with clear edge between cyst and host tissue. CE causes mortality in very few patients and there is a relatively good prognosis after surgical removal of the cystic lesion. Contrastingly, AE causes severe damage to the liver and patients require extensive treatment with albendazole to prevent relapse. However, little is known about the molecular mechanisms underpinning biological differences between the two parasites and the diseases they cause.

The differential expression of these KDPI genes between *E. granulosus* *s.s.* and *E. multilocularis* may be associated with the differences in pathology caused by the metacestodes of the two species. It would be informative to determine whether these genes play a role in determining the different pathologies resulting from infection by the two cestodes in their intermediate hosts.

Signal peptide analysis showed that 89.47% of *E. multilocularis* KDPIs contain a signal peptide compared to only 60.87% of *E. granulosus* *s.s.* KDPIs containing signal peptide sequences. It may indicate that the proportion of secretion protein in *E. multilocularis* KDPIs is relatively higher than that of *E. granulosus* *s.s.* KDPIs. It is not known whether the high percentage KDPIs of *E. multilocularis* containing signal peptides is associated with the virulent pathology of AE lesion.

*E. granulosus* *s.s.* has 5 genes EG_07242, EG_07266, EG_07243, EG_09006 and EG_09008, that *E. multilocularis* does not have. Whereas, these two genes, EmuJ_001136700.1 and EmuJ_001137100.1 are only existed in *E. multilocularis* genome. These differentially presented genes may play a role in the difference of pathology between the two parasites.

Gastrointestinal helminths survive in an environment containing proteases and these parasites must have mechanisms to control protease activation. Therefore, Kunitz domain inhibitors are important for parasite survival, especially intestinal dwelling helminth parasites, to counteract protease attack. Two *Echinococcus* stages, the oncosphere and adult worm, are found in the gastrointestinal duct. The oncosphere is activated in the stomach and penetrates through the intestinal wall before being passed into the internal organs, whereas the adult worm spends its whole life in the gastrointestinal duct which contains high concentrations of proteases such as pepsin, trypsin and chymotrypsin. We previously showed that two KDPIs, EgKI-1(EG_08721) and EgKI-2 (EG_7242) function as protease inhibitors. EgKI-1 (also has accession number EUB56407.1) is highly expressed in the oncosphere and EgKI-2 (GenBank: EUB57880.1) is highly expressed in the adult worm [14]. These KDPIs are differentially expressed and stage-specifically protect *E. granulosus* *s.s.* from protease attack [13]. In this study, we showed that 11 out 23 Eg-KDPIs were highly expressed in adult worms. These Eg-KDPIs likely protect against protease attacks in the gut during adult worm development. EG_05483 and EG_08721 were relatively highly expressed in oncospheres, suggesting their expressed products might be potential vaccine candidates against primary infection in the intermediate hosts of *E. granulosus s.s. .*

We did not find any KDPIs that were differentially and highly expressed in protoscoleces in this study, although a previous study described a multigene family of eight (EgKU1-EgKU8) secreted Kunitz proteins from *E. granulosus* *s.s.* protoscoleces preferentially expressed by pepsin/H (+)-treated worms [15].

The secondary structures of proteins, especially the α-helix and β-strands play key roles in molecular function, cell stability, mechanical signaling, and tissue constitution as random coils are easily folded and exposed to the protein surface [26]. The basic structure of a Kunitz peptide domain contains a typical sequence with six highly conserved cysteine residues connecting three disulphide bridges (C1-C6, C2-C4 and C3-C5) which stabilizes the protein structure. Among the disulphide bridges, the C1-C6 and C3-C5 bridges are required for the maintenance of native confirmation [27],whereas the C2-C4 bond stabilizes the folded structure [28]. We found 10 sequences had lost the #2 cysteine, including 5 from *E. granulosus s.s. *, indicating no C2-C4 bridge in these proteins. The reduction of disulfide bonds may affect the stability of the protein. It is not known whether these 5 proteins formed different bridges impacting on the function of these KDPIs, indicating that these genes may have a different functional role.

Hydrophilicity analysis showed that the Em- and Eg-KDPIs have high hydrophobicity, which is a typical characteristic of membrane proteins. The transmembrane regions consist of 20 hydrophobic amino acids, which could have an anchoring effect on cell membranes.

We previously showed that EgKI-1 is highly expressed in the oncosphere, indicating this protein helps protect this stage from digestion by trypsin, chymotrypsin and pancreatic elastase before it penetrates the intestinal wall.

In this study, qPCR results showed that 3 out 4 KDPI genes of *E. multilocularis* homologous to those *E. granulosus* *s.s.* KDPIs were expressed in different pattern in three stages of this tapeworm including adult worm, protoscolex and cyst germinal membrane. It will be interesting to identify whether the expression of KDPIs is associated with the pathological difference in both intermediate hosts and definitive hosts of these two tapeworms.

It is predicted that EgKU-1 and EgKU-4 functionally blocks voltage-dependent potassium channels (Kv). The similar sequences of *E. multilocularis* EmuJ_001136700 and EmuJ_001137100(Additional file 8: Fig. S6), may likely function as a Kv blocker [29], which may be a clue for designing drug targets against alveolar echinococcosis. In addition, *Eudiplozoon nipponicum* EnKT1 (a KDPI) is an antihemorrhagic snake venom factor-like protein which exhibited a higher activity against plasmin and Factor Xa which can act as a C3 and C5 convertase and impaired both haemostasis and complement activation [30]. The different expression of similar KDPIs in *E. granulosus s.s. and E. multilocularis* may be associated with the difference in cellular pathology of these two echinococcal diseases. It needs to be uncovered in future studies.

It is shown that parasite KDPIs with immunomodulatory activity can be vaccine candidates. *Fasciola hepatica* FhKTM decreased dendritic cell activation and may be involved in the immune evasion mechanisms of the parasite [21]. FhKTM induced mice protection against *F. hepatica* challenge by preventing liver damage and improving survival, likely through eliciting potent IFN-gamma and IL-17 A with high levels of antigen-specific IgG1, IgG2a, and IgA serum antibodies [31]. The KDPI EGR_05316 of *E. granulosus* *s.s.* has sililar sequences with Smp_147730 of *S. mansoni* [12, 32]. Two KDPIs EGR_05483 and EmuJ_000077800.1 of *E. granulosus* *s.s.* and *E. multilocularis* have similar sequences with FhKT1.1, FhKT1.2 and FhKT2 of F. hepatica [33] (Additional file 7: Fig. S5).

A study showed that *Schistosoma mansoni* Kunitz peptides were highly protective in vaccinated BALB/c mice in terms of reduction in recovery of adult females (89~91%) and in the numbers of eggs trapped in the livers (77~81%) and guts (57~77%)of mice [32]. In addition, SmKI-1 showed 23~33% of reductions in adult worm [13]. EGR_03481, EGR_07242 and EGR_05316 of *E. granulosus* *s.s.* KDPIs have very similar sequences with Smp_147730 (Fig. 2), this indicates that these KDPIs of *Echinococcus* likely can be candidates for vaccination against echinococcosis.

## Conclusions

In conclusion, based on whole genome analysis, 19 and 23 Kunitz domain protease inhibitors were identified in *E. multilocularis* and *E. granulosus s.s. .* The differential expression of these KDPIs in different developmental stages of *E. granulosus s.s. * suggests that they may have different functions in regulation of host immune responses. The difference in characterization of KDPIs may be associated with the different pathology of metacestode stage of these two parasites. These should be further illuminated to determine their roles in echinococcal development and interface interactions between host and the tapeworms and such information may provide new insights for the prevention and treatment of cystic and alveolar echinococcosis.

## Materials and methods

### Identification of *E. granulosuss.s.* and *E. multilocularis* Kunitz domain sequences

The *E. granulosus* *s.s.* and *E. multilocularis* genomes were previously completed by the Chinese National Human Genome Centre in Shanghai (CHGC) and the Wellcome Sanger Institute, United Kingdom in 2013 [18, 34]. Genome data is available from http://www.sanger. ac.uk/resources/downloads/helminths/ (*E. multilocularis, E. granulosus*). The complete genome annotation is available at www.genedb.org.

*E. granulosus s.s. , E. multilocularis* and *Fasciola hepatica* sequence data reported in this manuscript are accessible at WormBase ParaSite and the corresponding accession numbers indicated in Additional file 9: Table S3. *E. multilocularis* data have been deposited in the National Center for Biotechnology Information (NCBI) under the project accession number PRJEB122 and their Genome Assembly accession number is GCA_000469725.3. *E. granulosus* *s.s.* data have been deposited in NCBI (BioProject numbers: PRJEB121 and PRJNA182977) and their Genome Assembly accession numbers were GCA_000469785.1 and ASM52419v1. Based on the DNA genomic sequences, 11,325 and 10,429 genes were predicted for *E. granulosus s.s. *, and *E. multilocularis*, respectively. The InterProScan program (https://www.ebi.ac.uk /interpro/result/Inter- ProScan/) and Motif scan (https://myhits.sib.swiss/cgi-bin /motif_scan) were used to identify the Kunitz domain protease inhibitor (KDPI) sequences.

### Physiological/biochemical characters

The physiological/biochemical characters of KDPIs including molecular weight, isoelectric point and instability index were predicted using the ProtParam online software (http://web.Expasy.org/protparam/). Signal peptides were predicted with the SignalP 5.1 Server (http://www.cbs.dtu.dk/services/SignalP/). Post-translational modification sites were identified by MotifScan (http://hits.isb-sib.ch/cgi-bin/ motif_scan/).

The conservative structural domain of each KDPI was predicted using the Conserved Domain program (http://www.ncbi.nlm.nih.gov/cdd/); their subcellular localization was predicted using ProtCompv. 9.0 (http://linux1.softberry.com/berry. phtml?topic=protcompan&group=programs&subgroup=proloc/). Transmembrane regions were predicted by TMPred (http://embnet.vital-it.ch/cgibin/TMPRED_ form_parser) and TopPred 1.10 (http://mobyle.pasteur.fr/cgi-bin/portal.py?# forms::toppred). The hydrophilicity plot was predicted by ProtScale (http://web.expasy.org/protscale/). The secondary structures of KDPIs were predicted using SOPMA (https://npsa-prabi.ibcp.fr/cgi-bin/npsa_automat.pl?page=npsa_ sopma/). These genes were annotated to the Gene Ontology (GO) database for biological process (BP), molecular function (MF), and cellular component (CC) using Blast2GO PRO (https://www.blast2go.com/).

Three-dimensional (3D) structures of KDPIs were constructed using the automated modeling program within the online service SWISS-MODEL. The 3D models of KDPIs were assessed by Verify_3D (http://services.mbi.ucla.edu/Verify_3D/).

The multiple sequence alignment was analyzed and ordered by Clustal omega (http://www.ebi.ac.uk/Tools/msa/clustalo/) and then visually edited with BioEdit software v7.1.3. The phylogenetic tree was constructed by MEGA version 7 (http://www.megasoftware.net/) and Interactive Tree of Life iTOL v6 (https://itol.embl.de) with the maximum likelihood method. Bootstrapping analysis was performed and the bootstrap values which display support values (1000 bootstraps) are shown on the nodes. The clades are represented by different color. The complete KDPI protein sequences of single domain KDPIs were used for phylogenetic tree analysis.

### Expression of Kunitz domain inhibitors in *E. granulosuss.s.* and *E. multilocularis*

Transcript reads were obtained for each of the KDPI genes expressed in the adult worm, oncosphere, protoscolex and cyst (cyst germinal membrane) of *E. granulosus* *s.s.* using Hiseq techniques as described [18]. Based on these data, the expression of 8 KDPI genes from the *E. multilocularis* and *E. granulosus* *s.s.* expression was validated for adult worm, protoscolex and cyst stages using quantitative reverse transcription PCR (qRT-PCR).

### Total RNA extraction and cDNA synthesis

*E. granulosus* *s.s.* protoscoleces were collected from the cysts of sheep livers collected in slaughterhouses in Urumqi, Xinjiang, China. *E. multilocularis* protoscoleces were obtained by Mongolia gerbils maintained in the animal laboratory of the First Affiliated Hospital of Xinjiang Medical University. Adult worms of *E. granulosus* *s.s.* (35 days-old) and *E. multilocularis* (25 days-old) were provided by the Institute of Veterinary Research, Xinjiang Academy of Animal Sciences.

Isolated protoscoleces were washed 10 times with saline and stored at -80 ℃ until used. TRIzol reagent (Invitrogen, USA) was used for extraction of total RNA according to the manufactory’s instructions. RNA concentration was determined by a spectrophotometer (NanoDrop 2000, Thermo Scientific, USA) at 260 nm and the purity of RNA was considered satisfactory if the ratio of absorbance at 260 nm and 280 nm(A260/280) is ranged from 1.9 to 2.0.

Total RNA was reverse transcribed into cDNA using Reverse Transcriptase Kit (Takara, Dalian, China) following the instructions of manufacturer. The cDNAs were stored at −80 °C until used.

### Quantitative PCR (qPCR)

Gene-specific primers were designed with Primer 3 software and listed in Table 4, *eif*3 was used as the internal reference [35].The qPCR reactions were carried out in a CFX96 Real-Time PCR Detection System (Bio-Rad, USA). Experiments were performed with QuantiNova SYBR Green PCR Kit (QIAGEN). Each individual sample was run in triplicate. The qPCR cycling reactions were initially denatured at 95 °C for 2 min followed by 39 cycles of 95 °C for 5 s, 60 °C for 30 s. The melting curve analysis of 5 s per step from 65 to 95 °C after amplification was conducted to assess primer specificity. The mRNA expression level of the target cytokines relative to the reference gene were analyzed using the 2^−ΔΔCt^ method [36].

**Table 4:** Sequences of forward and reverse primers for the genes analysed by qPCR

Gene	Primer sequences(5’-3’)	Product size
EGR_08716	F: CCAATCTCGCACTTCTACTCCTCATG R: ATCGACAGGGTCCTACCTCAATGG	102 bp
EGR_07244	F: TCCTCCTCGTGGTCATCAGCTATTC R: CCACAACCGCCGTAGATGAACTC	148 bp
EGR_07944	F: AAGTGCCTTCATCGCCTCCATTG R: CCGCCTCTGATTCTGCTTTCTCTG	96 bp
EGR_09490	F: CCAGTCATAGCGGGCATCAGTTG R: GTCATCGTCTCCGTGGCATTCC	113 bp
EmuJ_001137000	F: CTCCTCATGCTGCTCGGTGTTG R: ATCGACAGGGTCCTACCTCAATGG	85 bp
EmuJ_000534800	F: TTTCCTCCTTGTGGTCATCAGCTATTC R: CACAACCGCCGTAGATGAACTCC	149 bp
EmuJ_000302900	F: TACAATGGTCACGGCGGCAATG R: TCTGGCGTTCTGGCACAAATCG	99 bp
EmuJ_000929500	F: GCACATCTTCAGTGGCAGGTATCC R: TGGCGGTTGGCATTTGGACTAC	87 bp
Eif3	F: GTTACATCCCTCCGACCTTG R: AAGCAGCCTCCTCTTGAGTG	243 bp

Note: F, forward primer; R, reverse primer

### Statistical analysis

Data are presented as means or median. All data are presented as the means± standard deviation (SD) of three individual experiments unless otherwise stated. Group comparisons were assessed by Two-tailed Student’s t test, Mann-Whitney U test and one-way analysis of variance (ANOVA) for statistically significant differences using GraphPad Prism software (Version 8.01). Chi square test followed by Fisher’s Exact Test was used to compare the sample rate (or constituent ratio) between the two groups. *P v*alues of <0.05 was considered significant in statistical analysis. (* *P* value *≤* 0.05; ** *P* value *≤* 0.001; *** *P* value *≤* 0.0001).

## Supplementary Information

**Additional file 1:** **Table S1. **Physiological andbiological characteristics of each of *E.granulosus* *s.s.* and *E. multilocularis *KDPIs.

**Additional file 2: Table S2.** The cellular localization of the *E. multilocularis* and *E. granulosus s.s. *KDPIs.

**Additional file 3: Figure S1. **Structure and amino acidcomposition of a Kunitz-domain peptide.

**Additional file 4: Figure S2. **Three dimensional structures of multi-domain Kunitz protease inhibitors in* E.granulosus s.s. *and* E. multilocularis* using SWISS-MODEL.

**Additional file 5: Figure S3. **Species-specific sequence alignment of  *E.granulosus s.s. *and* E.multilocularis* KDPIs genes.

**Additional file 6: Figure S4. **Phylogram constructed using the maximum likelihood method to compare EGR_03480 of  *E. granulosus s.s. *with the KDPIs from bovine, humans and other species.

**Additional file 7: Figure S5. **Phylogenetic analysisof *E. granulosus s.s. , E. multilocularis *and *Fasciola hepatica *Kunitz-type inhibitors.

**Additional file 8: Figure S6.** Sequence alignment of *E. granulosus s.s. *KU1~8 and Corresponding homologous* E. multilocularis* KDPIs genes.

**Additional file 9:** **Table S3. **The assembly accession numbers of the *E.multilocularis*, *E. granulosus s.s.,* *Fasciolahepatica* and* Schistosomamansoni* KDPIs

## Data Availability

All data generated or analyzed during this study are included in this published article and the additional data file.
