# Supplementary material for: Bioinformatic comparison of Kunitz protease inhibitors in Echinococcus granulosus sensu stricto and E. multilocularis and the genes expressed in different developmental stages of E. granulosus s.s
Source: BMC Genomics. 2021 Dec 18;22:907. doi: 10.1186/s12864-021-08219-4 (PMC8684439; doi:10.1186/s12864-021-08219-4)
Supplement: Supplementary file 1 — Additional file 1: Table S1. Physiological andbiological characteristics of each of E.granulosus s.s. and E. multilocularis KDPIs. [file 12864_2021_8219_MOESM1_ESM.docx]

**Table S1** Physiological and biological characteristics of Kunitz protease inhibitors in *E. granulosus* and *E. multilocularis*

| **Accession number** | **No. of aa** | **Molecular weight (Da)** | **Isoelectric points** | **No. of transmembrane** | **No. of cysteine in Kunitz domain** | **No. of cysteine in the protein** | **Instability index** | **Stable protein** | **Aliphatic index** | **Grand average of hydropathicity** | **Signal peptide** | **No. of Kunitz Motifs** | **P1 residue** |
| --- | --- | --- | --- | --- | --- | --- | --- | --- | --- | --- | --- | --- | --- |
| EG_01779 | 878 | 97357.81 | 7.13 | 0 | 6 | 61 | 59.98 | NO | 60.11 | -0.423 | NO | 4 | S |
| EG_03480 | 88 | 9948.5 | 8.26 | 0 | 6 | 6 | 46.17 | NO | 53.3 | 0.159 | Yes | 1 | R |
| EG_03481 | 242 | 27250.36 | 9.21 | 0 | 6 | 9 | 43.59 | NO | 72.52 | -0.468 | NO | 1 | F |
| EG_04958 | 135 | 15973.24 | 8.83 | 0 | 6 | 9 | 54.73 | NO | 56.37 | -0.535 | NO | 1 | R |
| EG_05316 | 239 | 25622.41 | 9.53 | 1C-T | 6 | 8 | 32.22 | Yes | 81.34 | -0.047 | NO | 1 | A |
| EG_05317 | 1540 | 168625.8 | 5.68 | 0 | 6 | 159 | 42.51 | NO | 54.55 | -0.467 | NO | 2 | L |
| EG_05482 | 130 | 15265.07 | 4.52 | 0 | 4 | 4 | 77.57 | NO | 69 | -0.652 | Yes | 1 | D |
| EG_05483 | 191 | 21013.71 | 8.72 | 2N-T | 4 | 6 | 46.5 | NO | 100.73 | 0.371 | NO | 1 | A |
| EG_07242 | 83 | 9290.6 | 8.28 | 0 | 5 | 6 | 45.87 | NO | 62.53 | -0.382 | Yes | 1 | R |
| EG_07243 | 75 | 8407.68 | 6.8 | 0 | 6 | 6 | 35.97 | Yes | 61.2 | -0.017 | Yes | 1 | R |
| EG_07244 | 106 | 11883.91 | 5.26 | 1 N-T | 6 | 8 | 31.97 | Yes | 87.45 | 0.192 | NO | 1 | L |
| EG_07266 | 129 | 14430.69 | 5.34 | 1 C-T | 6 | 5 | 42.85 | NO | 90.85 | 0.287 | Yes | 1 | L |
| EG_07944.1 | 539 | 61611.14 | 4.68 | 0 | 4 | 18 | 70.41 | NO | 49.44 | -0.996 | NO | 1 | E |
| EG_08716 | 84 | 9205.69 | 9.01 | 0 | 6 | 7 | 47.38 | NO | 77.86 | -0.075 | Yes | 1 | R |
| EG_08718 | 144 | 16174.44 | 8.07 | 0 | 6 | 9 | 31.85 | Yes | 73.75 | -0.214 | NO | 1 | R |
| EG_08720 | 84 | 9335 | 9.36 | 0 | 6 | 7 | 38.38 | Yes | 84.76 | 0.111 | Yes | 1 | R |
| EG_08721 | 79 | 8897.44 | 8.2 | 0 | 6 | 7 | 28.32 | Yes | 91.39 | -0.014 | Yes | 1 | L |
| EG_09006 | 89 | 10130.89 | 9.3 | 0 | 6 | 6 | 55.15 | NO | 80 | -0.354 | Yes | 1 | R |
| EG_09007 | 86 | 9535.37 | 9.71 | 0 | 6 | 7 | 31.25 | Yes | 80.58 | -0.122 | Yes | 1 | G |
| EG_09008 | 102 | 11848.18 | 10.52 | 0 | 6 | 8 | 46.53 | NO | 70.78 | -0.501 | Yes | 1 | T |
| EG_09269 | 92 | 10924.74 | 9.71 | 1 N-T | 6 | 7 | 35.52 | Yes | 63.59 | -0.203 | Yes | 1 | R |
| EG_09490 | 976 | 108775.1 | 8.79 | 0 | 5 | 46 | 50.29 | NO | 62.67 | -0.529 | Yes | 1 | N |
| EG_10096 | 98 | 11183.59 | 5.53 | 0 | 6 | 8 | 94.41 | NO | 56.84 | -0.247 | Yes | 1 | R |
| EmuJ_000077700.1 | 210 | 24220.2 | 5.68 | 0 | 4 | 4 | 62.44 | No | 71.9 | -0.657 | yes | 1 | D |
| EmuJ_000077800.1 | 136 | 15116.53 | 6.53 | 1 N-T | 4 | 5 | 47.86 | No | 89.78 | 0.152 | No | 1 | A |
| EmuJ_000225800.1 | 1292 | 144231.47 | 5.6 | 0 | 5 | 85 | 59.61 | No | 59.59 | -0.481 | yes | 5 | T |
| EmuJ_000255800.1 | 2425 | 266767.88 | 5.16 | 1 N-T | 6 | 217 | 45.41 | No | 55.76 | -0.476 | yes | 5 | K |
| EmuJ_000302900.1 | 534 | 61174.77 | 4.71 | 0 | 4 | 18 | 67.41 | No | 51.54 | -0.978 | yes | 1 | E |
| EmuJ_000419200.1 | 92 | 10929.84 | 9.84 | 1 N-T | 6 | 8 | 43.59 | No | 56.2 | -0.21 | yes | 1 | R |
| EmuJ_000534800.1 | 75 | 8337.74 | 5.22 | 0 | 6 | 6 | 32.38 | Yes | 70.27 | 0.023 | yes | 1 | L |
| EmuJ_000548800.1 | 99 | 11242.72 | 6.03 | 0 | 6 | 8 | 90.66 | No | 56.26 | -0.194 | yes | 1 | R |
| EmuJ_000549400.1 | 100 | 11738.43 | 7.53 | 0 | 6 | 7 | 55.02 | No | 57.6 | -0.301 | yes | 1 | R |
| EmuJ_001136500.1 | 89 | 9910.42 | 5.24 | 0 | 6 | 7 | 26.51 | Yes | 87.64 | 0.01 | yes | 1 | L |
| EmuJ_001136600.1 | 84 | 9369.1 | 9.33 | 0 | 6 | 7 | 37.63 | Yes | 88.21 | 0.092 | yes | 1 | R |
| EmuJ_001136700.1 | 78 | 8763.29 | 9.18 | 0 | 6 | 8 | 29.79 | Yes | 83.72 | -0.228 | yes | 1 | Q |
| EmuJ_001136800.1 | 89 | 9910.42 | 5.24 | 0 | 6 | 7 | 26.51 | Yes | 87.64 | 0.01 | yes | 1 | L |
| EmuJ_001136900.1 | 90 | 10093.88 | 9.56 | 0 | 6 | 7 | 42.78 | No | 77 | -0.362 | yes | 1 | R |
| EmuJ_001137000.1 | 84 | 9148.63 | 9.33 | 0 | 6 | 6 | 45.8 | No | 77.86 | -0.12 | yes | 1 | R |
| EmuJ_001137100.1 | 84 | 9596.51 | 10.17 | 0 | 6 | 8 | 30.44 | Yes | 74.17 | -0.463 | yes | 1 | R |
| EmuJ_001137300.1 | 80 | 9211.98 | 9.6 | 0 | 6 | 8 | 41.39 | No | 88.88 | 0.34 | yes | 1 | S |
| EmuJ_001137400.1 | 85 | 9393.2 | 9.6 | 0 | 6 | 7 | 28.66 | Yes | 82.71 | -0.045 | yes | 1 | G |
| EmuJ_001181950.1 | 610 | 66753.7 | 7.9 | 1 N-T | 6 | 50 | 44.38 | No | 58.07 | -0.335 | No | 8 | K |

Note: E.m, *E. multilocularis*; E.g, *E. granulosus*; KDPIs, Kunitz domain protease inhibitors; No. of aa, number of amino acids; No. of tran-domain, number of transmembrane domains; Aver of cysteine, average of cysteine per sequence; Aliphatic indexes; GRSVY, hydropathic index; En-targ (T/C), enzyme targeting inhibitors, trypsin inhibitors(T) or chymotrypsin inhibitors(C).
