# Supplementary material for: Bioinformatic comparison of Kunitz protease inhibitors in Echinococcus granulosus sensu stricto and E. multilocularis and the genes expressed in different developmental stages of E. granulosus s.s
Source: BMC Genomics. 2021 Dec 18;22:907. doi: 10.1186/s12864-021-08219-4 (PMC8684439; doi:10.1186/s12864-021-08219-4)
Supplement: Supplementary file 2 — Additional file 2: Table S2. The cellular localization of the E. multilocularis and E. granulosus s.s. KDPIs. [file 12864_2021_8219_MOESM2_ESM.docx]

**Table S2** The cellular localization of the *E. multilocularis* and *E. granulosus* KDPIs

| **Accession** | **GO ids** | **GO terms** | **Score** | **Signal Peptide** |
| --- | --- | --- | --- | --- |
| EmuJ_000077700.1 | GO:0005615 | C: extracellular space | 0.98 | 1-19 |
| EmuJ_000077800.1 | GO:0005615 | C: extracellular space | 1 | / |
| EmuJ_000225800.1 | GO:0005615 | C: extracellular space | 1 | 1-26 |
| EmuJ_000255800.1 | GO:0005615 | C: extracellular space | 0.9 | 1-25 |
| EmuJ_000302900.1 | GO:0005615 | C: extracellular space | 0.99 | 1-20 |
| EmuJ_000419200.1 | GO:0005615 | C: extracellular space | 0.92 | 1-20 |
| EmuJ_000534800.1 | GO:0005615 | C: extracellular space | 1 | 1-18 |
| EmuJ_000548800.1 | GO:0005615 | C: extracellular space | 1 | 1-20 |
| EmuJ_000549400.1 | GO:0005615 | C: extracellular space | 1 | 1-18 |
| EmuJ_001136500.1 | GO:0005615 | C: extracellular space | 1 | 1-20 |
| EmuJ_001136600.1 | GO:0005615 | C: extracellular space | 1 | 1-20 |
| EmuJ_001136700.1 | GO:0005615 | C: extracellular space | 1 | 1-20 |
| EmuJ_001136800.1 | GO:0005615 | C: extracellular space | 1 | 1-20 |
| EmuJ_001136900.1 | GO:0005615 | C: extracellular space | 1 | 1-18 |
| EmuJ_001137000.1 | GO:0005615 | C: extracellular space | 1 | 1-20 |
| EmuJ_001137100.1 | GO:0005615 | C: extracellular space | 1 | 1-20 |
| EmuJ_001137300.1 | GO:0005615 | C: extracellular space | 1 | 1-20 |
| EmuJ_001137400.1 | GO:0005615 | C: extracellular space | 1 | 1-20 |
| EmuJ_001181950.1 | GO:0005737 | C: cytoplasm | 0.7 | / |
| EG_01779 | GO:0005737 | C: cytoplasm | 0.7 | / |
| EG_03480 | GO:0005615 | C: extracellular space | 0.99 | 1-20 |
| EG_03481 | GO:0005737 | C: cytoplasm | 0.7 | / |
| EG_04958 | GO:0005615 | C: extracellular space | 0.8 | / |
| EG_05316 | GO:0005737 | C: cytoplasm | 0.7 | / |
| EG_05317 | GO:0005615 | C: extracellular space | 1 | / |
| EG_05482 | GO:0005615 | C: extracellular space | 1 | 1-19 |
| EG_05483 | GO:0012505 | C: endomembrane system | 0.35 | / |
| EG_07242 | GO:0005615 | C: extracellular space | 1 | 1-18 |
| EG_07243 | GO:0005615 | C: extracellular space | 1 | 1-18 |
| EG_07244 | GO:0005886 | C: plasma membrane | 0.54 | / |
| EG_07266 | GO:0005615 | C: extracellular space | 0.81 | 1-19 |
| EG_07944.1 | GO:0005615 | C: extracellular space | 1 | / |
| EG_08716 | GO:0005615 | C: extracellular space | 1 | 1-20 |
| EG_08718 | GO:0005615 | C: extracellular space | 1 | / |
| EG_08720 | GO:0005615 | C: extracellular space | 1 | 1-20 |
| EG_08721 | GO:0005615 | C: extracellular space | 1 | 1-18 |
| EG_09006 | GO:0005615 | C: extracellular space | 1 | 1-20 |
| EG_09007 | GO:0005615 | C: extracellular space | 1 | 1-20 |
| EG_09008 | GO:0005615 | C: extracellular space | 1 | 1-20 |
| EG_09269 | GO:0005615 | C: extracellular space | 0.8 | 1-21 |
| EG_09490 | GO:0005615 | C: extracellular space | 0.9 | 1-20 |
| EG_10096 | GO:0005615 | C: extracellular space | 1 | 1-19 |
