# Supplementary figures and images for: Bioinformatic comparison of Kunitz protease inhibitors in Echinococcus granulosus sensu stricto and E. multilocularis and the genes expressed in different developmental stages of E. granulosus s.s
Source: BMC Genomics. 2021 Dec 18;22:907. doi: 10.1186/s12864-021-08219-4 (PMC8684439; doi:10.1186/s12864-021-08219-4)

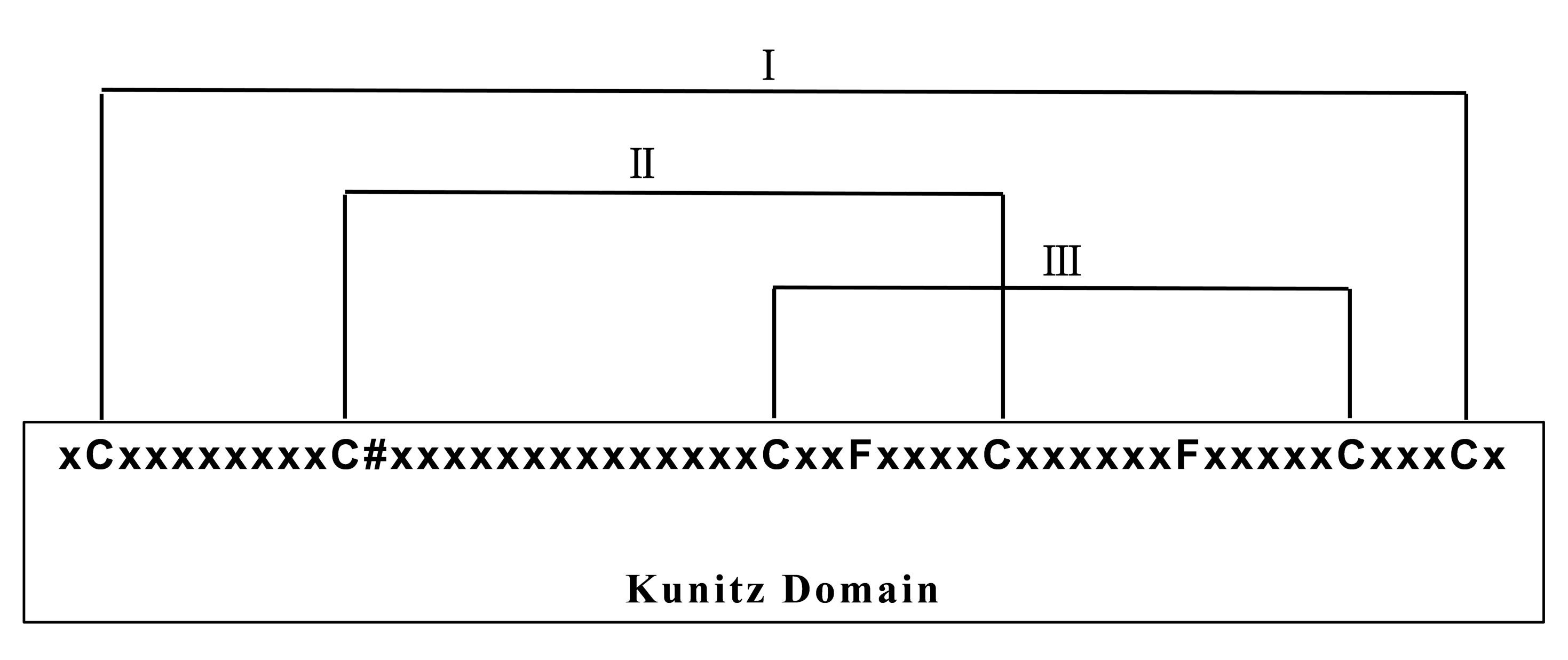

Supplement: Supplementary file 3 — Additional file 3: Figure S1. Structure and amino acidcomposition of a Kunitz-domain peptide. [file 12864_2021_8219_MOESM3_ESM.tif]

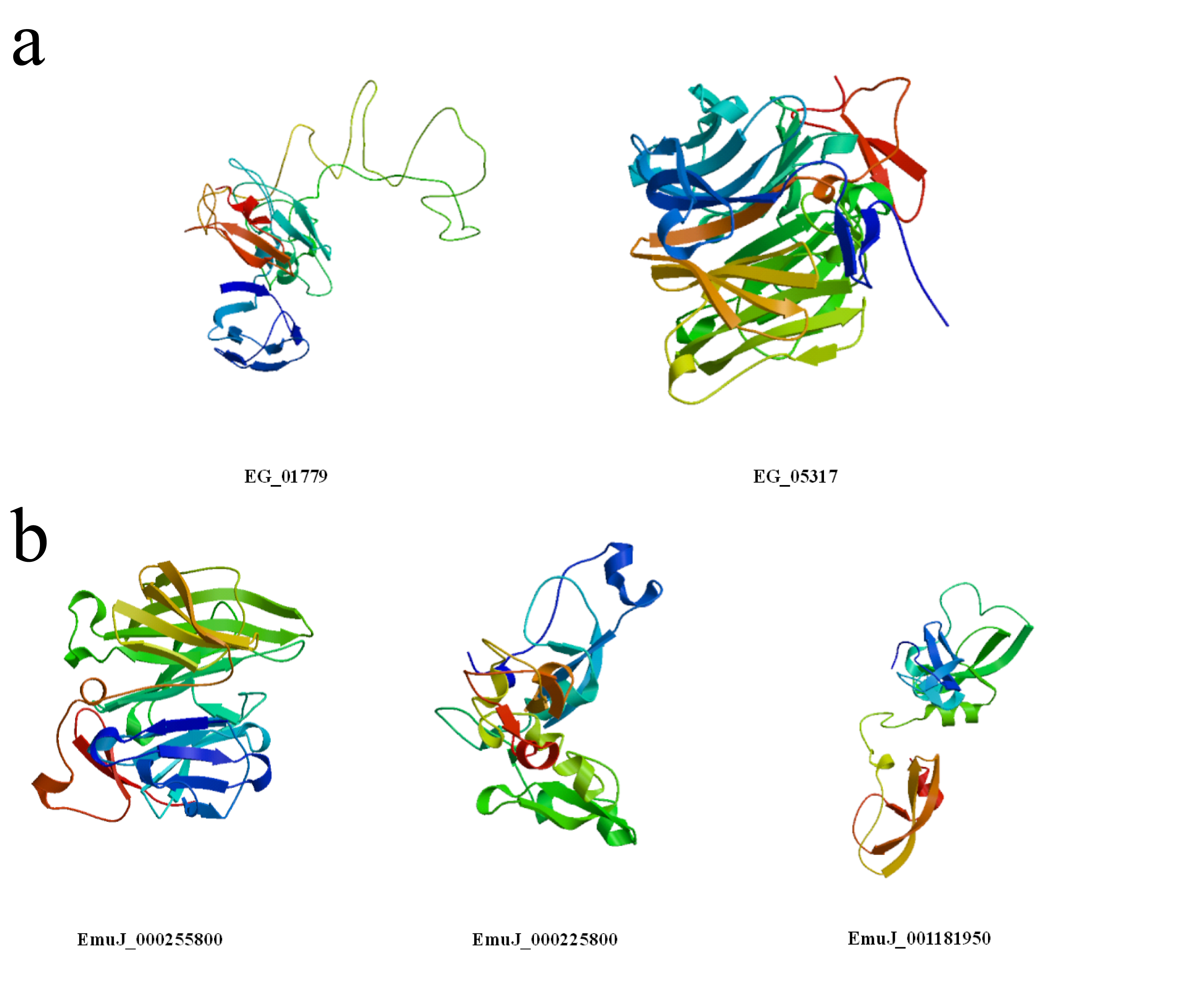

Supplement: Supplementary file 4 — Additional file 4: Figure S2. Three dimensional structures of multi-domain Kunitz protease inhibitors in E.granulosus s.s. and E. multilocularis using SWISS-MODEL. [file 12864_2021_8219_MOESM4_ESM.tif]

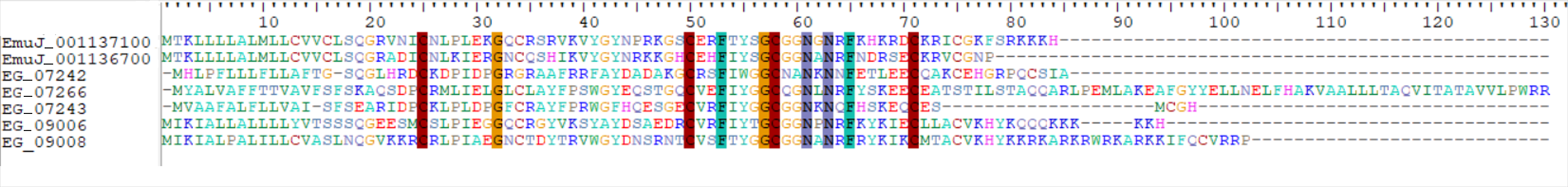

Supplement: Supplementary file 5 — Additional file 5: Figure S3. Species-specific sequence alignment of E.granulosus s.s. and E.multilocularis KDPIs genes. [file 12864_2021_8219_MOESM5_ESM.tif]

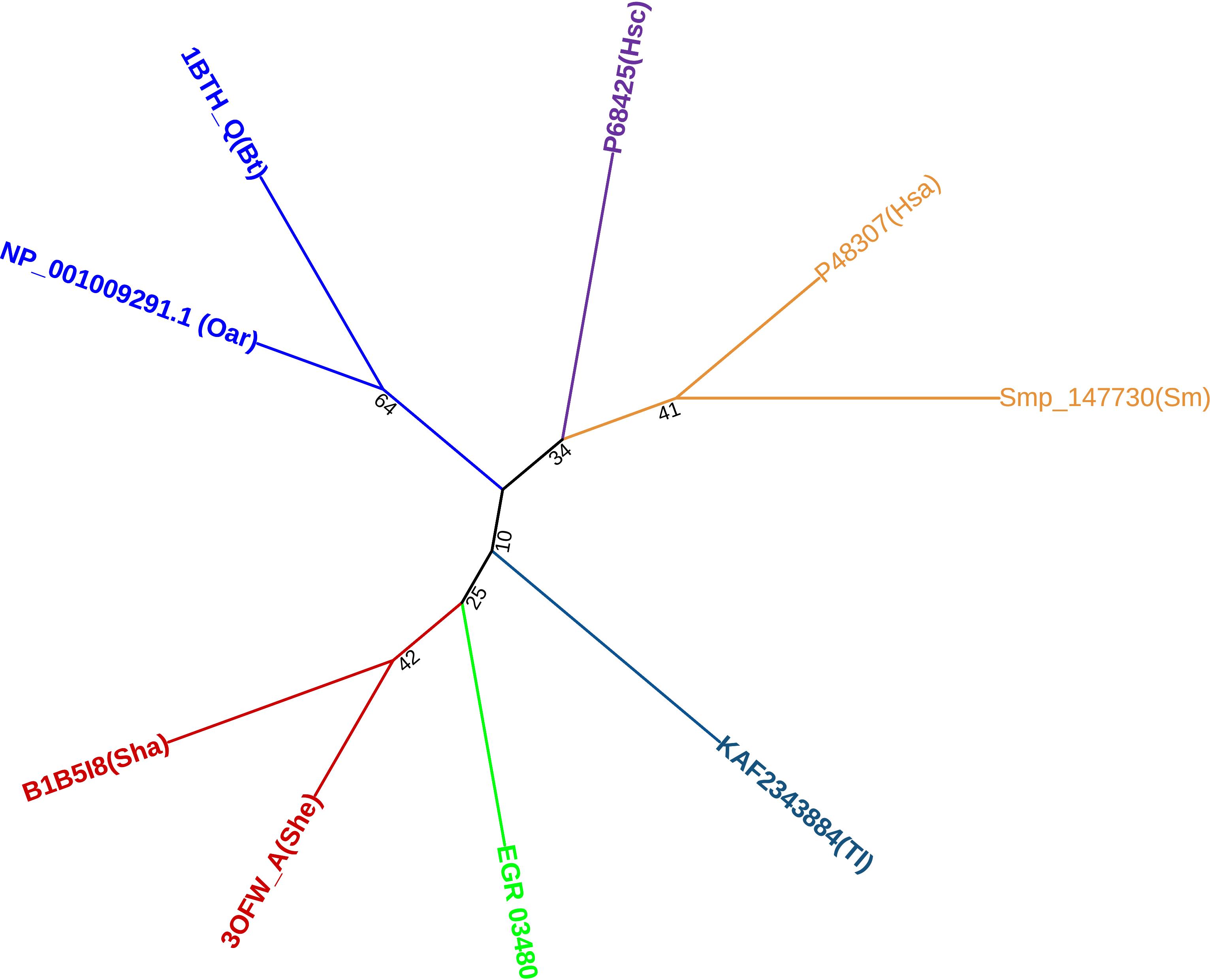

Supplement: Supplementary file 6 — Additional file 6: Figure S4. Phylogram constructed using the maximum likelihood method to compare EGR_03480 of E. granulosus s.s. with the KDPIs from bovine, humans and other species. [file 12864_2021_8219_MOESM6_ESM.jpg]

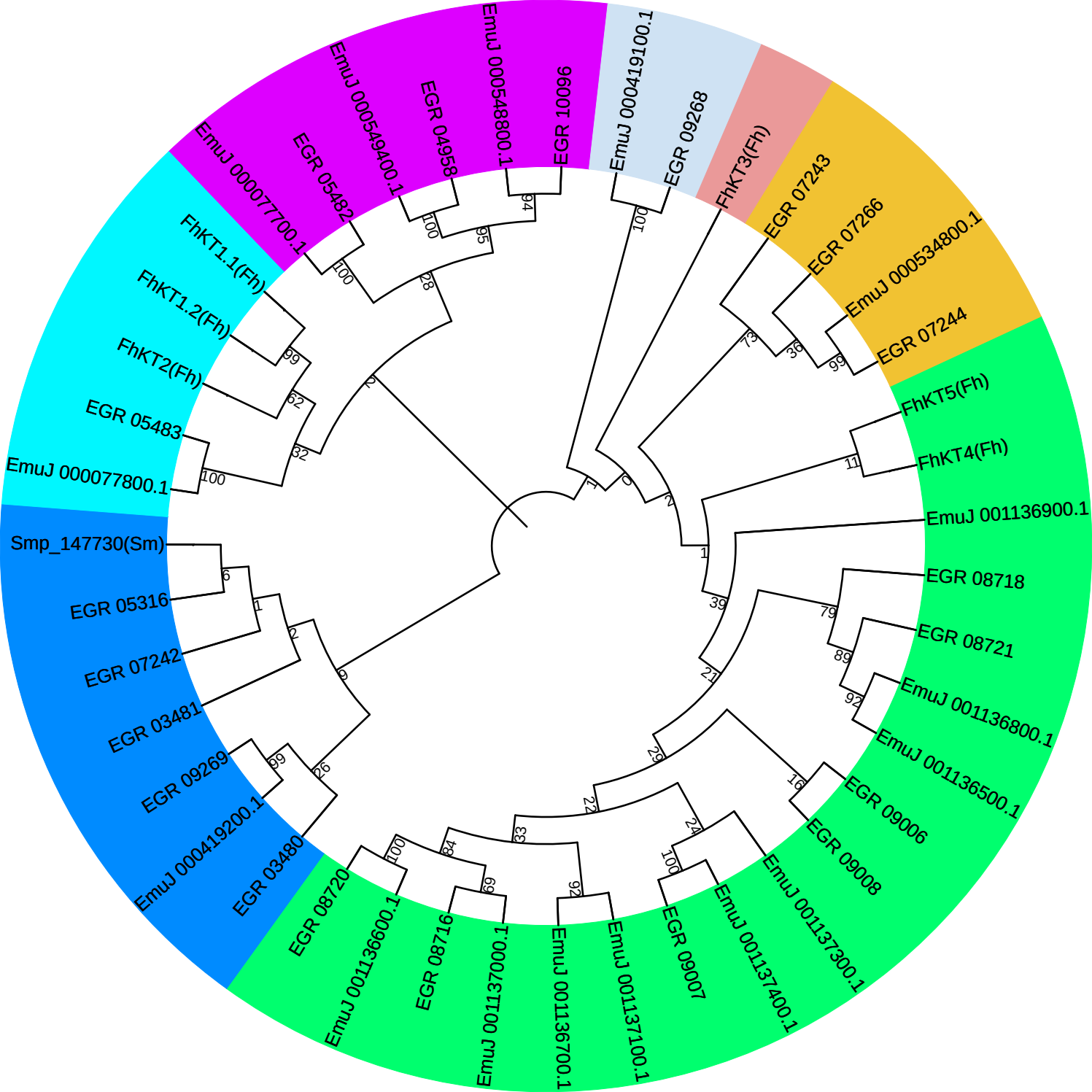

Supplement: Supplementary file 7 — Additional file 7: Figure S5. Phylogenetic analysisof E. granulosus s.s. , E. multilocularis and Fasciola hepatica Kunitz-type inhibitors. [file 12864_2021_8219_MOESM7_ESM.pdf]

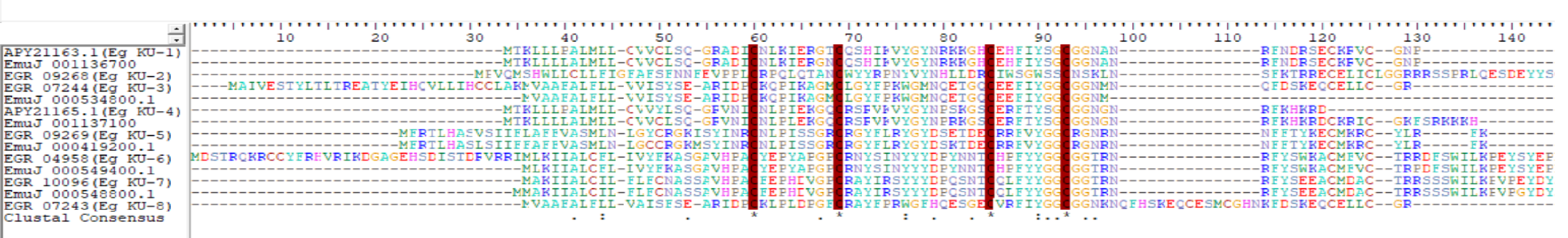

Supplement: Supplementary file 8 — Additional file 8: Figure S6. Sequence alignment of E. granulosus s.s. KU1~8 and Corresponding homologous E. multilocularis KDPIs genes. [file 12864_2021_8219_MOESM8_ESM.tif]
