# Supplementary material for: Bioinformatic comparison of Kunitz protease inhibitors in Echinococcus granulosus sensu stricto and E. multilocularis and the genes expressed in different developmental stages of E. granulosus s.s
Source: BMC Genomics. 2021 Dec 18;22:907. doi: 10.1186/s12864-021-08219-4 (PMC8684439; doi:10.1186/s12864-021-08219-4)
Supplement: Supplementary file 9 — Additional file 9: Table S3. The assembly accession numbers of the E.multilocularis, E. granulosus s.s., Fasciolahepatica and Schistosomamansoni KDPIs [file 12864_2021_8219_MOESM9_ESM.docx]

**Table S3** The assembly accession numbers of the *E. multilocularis,* *E. granulosus,* *Fasciola hepatica* and *Schistosoma mansoni* KDPIs

| **Taxonomy Name** | **BioProject Number** | **Genome Assembly Accession Number** | **BioSample** | **Submission Insitution** |
| --- | --- | --- | --- | --- |
| *Echinococcus multilocularis* | PRJEB122 | GCA_000469725.3 | SAMEA2271939 | Wellcome Trust Sanger Institute,  Wellcome Genome Campus |
| *Echinococcus granulosus* | PRJEB121 (EGRG)  PRJNA182977(EGR) | GCA_000524195.1  ASM52419v1 | [SAMN01914755](https://www.ncbi.nlm.nih.gov/biosample/SAMN01914755) | Wellcome Trust Sanger Institute,  Wellcome Genome Campus,  Chinese National Human Genome Center at Shanghai |
| *Fasciola hepatica* | PRJEB25283  PRJNA179522  [PRJEB6687](https://www.ncbi.nlm.nih.gov/bioproject/PRJEB6687) | GCA_900302435.1  GCA_002763495.1  [GCA_000947175.1](https://www.ncbi.nlm.nih.gov/assembly/GCA_000947175.1)  [GCA_000824725.2](https://www.ncbi.nlm.nih.gov/assembly/GCA_000824725.2) | [SAMEA104656475](https://www.ncbi.nlm.nih.gov/biosample/SAMEA104656475)  [SAMN02780903](https://www.ncbi.nlm.nih.gov/biosample/SAMN02780903)  [SAMEA2629804](https://www.ncbi.nlm.nih.gov/biosample/SAMEA2629804) | University of Liverpool,  [The Genome Institute](http://genome.wustl.edu/) |
| *Schistosoma mansoni* | PRJEA36577 | GCA_000237925.3 | SAMEA2272516 | Wellcome Trust Sanger Institute |
